# Standardization and quality control in quantifying non-enzymatic oxidative protein modifications in relation to ageing and disease: Why is it important and why is it hard?

**DOI:** 10.1016/j.redox.2015.04.001

**Published:** 2015-04-14

**Authors:** Olgica Nedić, Adelina Rogowska-Wrzesinska, Suresh I.S. Rattan

**Affiliations:** a Institute for the Application of Nuclear Energy, University of Belgrade, Belgrade, Serbia; b Department of Biochemistry and Molecular Biology, Southern Denmark University, Odense, Denmark; c Department of Molecular Biology and Genetics, Aarhus University, Aarhus, Denmark

**Keywords:** Protein modification, Protein damage, Ageing, Health, Quantification, Standardization, Quality control

## Abstract

Post-translational modifications (PTM) of proteins determine the activity, stability, specificity, transportability and lifespan of a protein. Some PTM are highly specific and regulated involving various enzymatic pathways, but there are other non-enzymatic PTM (nePTM), which occur stochastically, depend on the ternary structure of proteins and can be damaging. It is often observed that inactive and abnormal proteins accumulate in old cells and tissues. The nature, site and extent of nePTM give rise to a population of that specific protein with alterations in structure and function ranging from being fully active to totally inactive molecules. Determination of the type and the amount (abundance) of nePTM is essential for establishing connection between specific protein structure and specific biological role. This article summarizes analytical demands for reliable quantification of nePTM, including requirements for the assay performance, standardization and quality control, and points to the difficulties, uncertainties and un-resolved issues.

## Highlights

• There are no reference materials for modified proteins except for glycated hemoglobin.
• Commercial non-enzymatically modified proteins exist, but they are rarely used.
• Non-enzymatically modified protein standards are mostly prepared “in house”.
• There is no official proficiency testing scheme, except for glycated hemoglobin.
• There are no criteria relating quantity of the modified protein and health assessment.

## Graphical abstract

**Figure:**
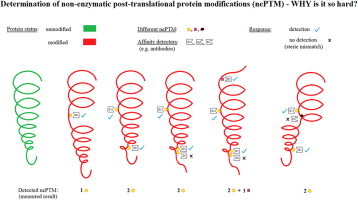

## Introduction

Common to all biological processes are molecular interactions, which include binding, followed by a cascade of reactions leading to specific physiological events. A cascade may consist of many binding interactions that obey a certain hierarchy such that the first event induces an ordered series of happenings that determines the so-called normal healthy state of a biological system. However, the biochemical processes that sustain life are prone to alterations owing to intrinsic and extrinsic factors and these changes underlie the emergence of ageing and diseases. Although most physiological macromolecules fit into a general binding-propagating pathway, this article deals with proteins, their interactions, effects of post-translational structural modifications, and the importance of standardization and quality control in the detection and measurement of molecular forms in physiological samples. Before analyzing difficulties in quantification of specific protein forms, we recapitulate some structural and binding properties of proteins, as follows.

## Folding and binding properties of proteins

All proteins exist as a population of many conformational states, known as conformers. Multiple conformers are derived from molecular motions that do not demand significant energy input, resulting in molecular states of similar stability [1]. Motions are predominantly at the level of side-chains, but the polypeptide backbone may also exert some flexibility. Although native protein conformation is dependent primarily on the amino-acid sequence, the surrounding environment contributes as well. The larger the molecule, the more conformers are likely to exist. Distribution of conformers is not even, as some are present in greater proportion and others in much smaller quantities. This dynamic population equilibrium shifts in the presence of a binding partner (ligand), which will “select” the favorable conformer (the one to interact with), causing redistribution of the conformer set. According to the concept of a pre-existing population of conformers [2], the ligand “chooses” the most suitable one from the “library” of offered molecules. The binding process further stabilizes the complex by optimizing protein folding and enabling new non-covalent interactions both within the protein and with the ligand. Along the cascade of interactions, each additional step induces conformational adjustments leading to multi-molecular complexes that will trigger specific physiological events.

Researchers use different words to express their understanding of molecular flexibility, introducing concepts such as intrinsic disorder, controlled chaos, conformational breathing or structural plasticity [3]. Proteins with a greater number of conformers exhibit more flexible ligand binding capacity, enabling interactions with a range of ligands [2]. These ligands may have homologous structure, but even dissimilar ligands may bind to the same site. The most typical examples are proteases and their substrates, which possess appropriate short amino-acid sequence regardless of the rest of the molecule [4]. Other examples are proteins involved in the innate immune response, where they recognize certain motives within a range of native molecules [5]. Antibodies bind epitopes which fit conformationally into Fab pockets, regardless of the appearance of the remaining molecule [6]. Computer modeling analysis has provided the information that proteins with a greater degree of conformational change utilize more interfaces for interactions [7].

## Post-translational modifications of proteins

Accurate translation of mRNA, followed by appropriate modifications of the polypeptide chain, is essential for normal folding, targeting and specificity. Misregulation in any of these steps can have far reaching biological consequences, including effects on cell growth, division and survival. Many post-translational modifications (PTM) of proteins have been described that determine their activity, stability, specificity, transportability and lifespan [8]. There are two distinct types of protein modification, one initiated (programmed) and catalyzed in the presence of specific enzymes (enzymatic modifications), and the other which occurs in the presence of compounds chemically reactive with proteins (e.g. reactive oxygen- and nitrogen-species) or is due to physical modification (e.g. irradiation). The second type of modification is known as non-enzymatic. Enzyme catalyzed PTM include phosphorylation, acetylation, glycosylation, ribosylation, methylation and some oxidation reactions such as formation of disulfide bonds and nitrosylation. Non-enzymatic post-translational modifications (nePTM) occur stochastically, depend on the ternary structure and can be physiologically damaging [9]. Such nePTM include oxidation, glycation, deamidation, racemization and isomerization.

Different reactive species are responsible for the formation of a range of oxidative products, while different amino-acid side chains can undergo modification contributing to an additional variety of derivatives. Some of these end-products are unstable, present in very low quantities and decompose quickly. The physiological response of an organism to nePTM proteins can be repair or degradation. Lysosomal or proteasomal degradation systems are primarily involved in the removal of damaged proteins [10], but cell surface receptors participate as well [11]. Other modified proteins acquire stability by further structural adjustments, which may end with misfolding. A misfolded protein has an increased chance of self-aggregation into insoluble fibrillar structures, which accumulate in cells and tissues. Fibrils display common cross-β sheets, regardless of the original protein structure. A strategy to reduce fibrillogenesis involves stabilization of the native structure, for example by binding an appropriate ligand [3]. That event would favor non-covalent inter-molecular interactions which do not encourage self-assembly [12]. Formation of amyloid fibrils underlines neurodegenerative disorders associated with ageing [13].

Proteins are not equally vulnerable to non-enzymatic modifications. In general, abundant and long living proteins have the most easily observed nePTM. Half-lives of soluble proteins are generally shorter than those of structural proteins, so the effects of modification may be limited and relatively small. If, however, conditions under which nePTM form last for a long period (i.e. permanent exposure to modifying agents), the consequences of even short-living derivatives may be serious. Besides half-life and concentration, the sequence and conformation of the protein contribute to its overall susceptibility to modification. Oxidation of fibrinogen, for example, occurs more readily than oxidation of albumin, immunoglobulins or transferrin [14]. Modifications of intracellular proteins induce functional metabolic changes. Proteins in mitochondria are subjected to oxidation more intensively than those in other compartments [15]. Oxidative changes of endothelial proteins are also common, underlying the pathogenesis of cardiovascular diseases [16]. Specific examples of modified proteins will be given further in the text, where appropriate.

## Accumulation of abnormal proteins during ageing

It is often observed that inactive and abnormal proteins accumulate in old cells and tissues [17], [18]. This increased amount of debris in the cytoplasm can be inhibitory for cell growth and normal metabolism, and thus contributes towards failure of maintenance. One reason for inactivation of an enzyme can be oxidative modification by oxygen free radicals, by mixed-function oxidation (MFO) systems or by metal catalyzed oxidation (MCO) systems. Since some amino-acid residues, particularly Pro, Arg, Thr and Lys, are oxidized to carbonyl derivatives, the carbonyl content of proteins has been used as an estimate of protein oxidation during ageing [19], [20].

An increase in the level of oxidatively modified proteins has been reported in old human erythrocytes of higher density and in cultured human fibroblasts from normal old donors and from individuals suffering from progeria and Werner’s syndrome [21], [22]. Similarly, there was a twofold increase in the carbonyl content of brain proteins of retired breeder Mongolian gerbils, which was reversed by treatment with the spin-trapping compound N-tert-butyl-phenylnitrone [23]. An age-related increase in the carbonyl content has also been reported in houseflies, fruitflies, nematodes and mouse organs [17], [18]. Declining activity of 6-phosphogluconate dehydrogenase and liver malic enzyme during ageing is related to the loss of Lys and His residues by oxidation. Oxidation of a Cys residue in glyceraldehyde-3-phosphate dehydrogenase may be responsible for its inactivation during ageing in rat muscles. Similarly, oxidatively-induced disulfide bridges in Cys and Met lead to the formation of sulfenic acid, sulfinic acid and methionine sulfoxide, which can accumulate during ageing [24]. It has also been reported that the concentration of oxidation products in human lens proteins and skin collagen increases along with the accumulation of oxidative forms of alpha-crystallin in patients with age-related cataract [25].

Structural alterations introduced into proteins by oxidation can lead to aggregation, fragmentation, denaturation and distortion of secondary and tertiary structure, thereby increasing the proteolytic susceptibility of oxidized proteins. Furthermore, toxic products of carbonyl modifications can react with other macromolecules and affect various metabolic processes. Oxygen, nitrogen and other reactive species can moderately change protein structure (mostly via interactions at side chains) or even cleave the protein into fragments (mostly via interactions at the backbone) [26], [27].

Glycation is another prevalent covalent modification in which the free amino groups of proteins react with glucose to form keto-amines, called Amadori products. This is followed by a sequence of further reactions and rearrangements producing the so-called advanced glycosylation end products (AGEs) [28]. Most commonly, it is the long-lived structural proteins, such as lens crystallins, collagen and basement membrane proteins, which are most susceptible to glycation. In the case of skin, vimentin has been found to be the main protein becoming glycated during ageing [29]. Glycated proteins are more prone to form cross-links with other proteins, leading to structural and functional alterations. Elevated levels of glycated proteins during ageing have been observed in a wide variety of systems [28]. For example, there is an increase in glycated Lys residues in rat sciatic nerve, aorta and skin collagen during ageing. Glycation of human collagen and osteocalcin is augmented during ageing. The formation and accumulation of AGEs are implicated in the physiology and pathology of senescence. It has been observed that pentosidine (cross-linked glycated Lys and Arg), carboxylmethyl-lysine (CML, glycated and oxidized proteins) and pyrroline increase with age in normal and diabetic humans [30]. By using AGE-specific antibodies, an AGE-modified form of human hemoglobin has been identified, the levels of which increase during ageing and in patients with diabetes-induced hyperglycemia [31], [32].

There is, however, a serious methodological limitation in assessing the significance of reported changes in the levels of nePTM proteins during ageing. Since there is an extremely low probability that any two molecules will be modified/damaged in exactly the same way and to the same extent, an increase in molecular heterogeneity is inevitable [18]. Furthermore, the nature, site and extent of damage will give rise to a population of a specific protein with alterations in structure and function ranging from being fully active to totally inactive molecules. Of course, among the thousands of types of proteins in a cell, some may be preferentially damaged in a particular context. For example, it has been reported that among 1000–2000 proteins inside mitochondria, aconitase is preferentially damaged oxidatively [33]. Some other proteins known to be prone to oxidation include Hsp70, protein elongation factors, glutamine synthetase, glutamate synthetase and pyruvate kinase. Similarly, vimentin is the specific target of glycation among thousands of other proteins in the skin. Therefore, accurate identification, detection and quantification of nePTM and their targets is essential for diagnosis and intervention in ageing and diseases.

## Quantification: why is it important?

Understanding the biochemistry and metabolism of modified proteins has gained increased attention in the last two decades, imposing a demand for their accurate detection and quantification. Since PTM are regular events which enable native proteins to perform specific normal and pathological physiological tasks, their determination contributes to recognition of a connection between specific protein structure and specific biological role. Analysis of nePTM covers two distinct features: (i) determination of the modification itself (i.e. chemical group or small entity), such as number of carbonyl groups, disulfide bonds, etc. and their specific location in the protein sequence and (ii) determination of the quantity of the specifically-modified protein, such as AGE-albumin or hemoglobin, in relation to the non-modified form and/or between two distinct conditions, e.g. healthy and diseased. Sometimes, these two aspects cannot be clearly separated.

As in the case with all other measurements, quantification of nePTM must follow the route already established for other analytes. The pattern may be summarized as follows:

• Development of suitable methods.
• Analytical characterization and validation of each method.
• Definition of standard preparations.
• Evaluation of the chosen standard(s) in different methods and modes of utilization.
• Definition of control samples.
• Evaluation of methods and their standardization on a large number of samples, obtained from appropriate reference populations: healthy young, old, male, female, lean, obese, meat-eaters, vegetarians, sedentary, involved in recreation, non-smokers, smokers – the choice of the reference population depends on the analyte to be determined.
• Evaluation of the method/standardization on samples obtained from patients with certain disease(s).
• Definition of a potential reference material (RM) from a library of standards tested.
• Inter-laboratory testing of unknown samples (ring trial), with participation of many laboratories.
• Data analysis.
• Consensus agreement.
• Definition of RM and reference method (maybe more than one).
• Assignment of RM production to a metrology institution.
• Periodical inter-laboratory testing, also known as proficiency testing.

The pattern described above will lead to harmonization in measurement procedures and traceability of the result value to reliable standard(s), fulfilling the general purpose of standardization in laboratory measurement, which is to achieve comparability of results obtained in different laboratories, at different times [34].

As already stated, the essential components of the standardization procedure are RMs and reference methods. The concept of metrological traceability, introduced as the principle standardization achievement, ensures that the test result is traceable to the appropriate RM [35]. The RM is a well-defined material, with the exact value, used to standardize the measurement assay. In contrast to the so-called control preparations, which are widely used in laboratories in every-day work and whose values are assigned consensus values (obtained after statistical evaluation of great number of determinations in many laboratories) the RM has a “true” value. A certain preparation becomes an RM after consensus agreement of the proficiency testing network of laboratories and it should be applicable for standardization of different methods. Production of an RM is assigned to a respectable metrology institution such as the National Institute of Standards and Technology (NIST), World Health Organization (WHO) or National Institute for Biological Standards and Control (NIBSC) [36]. The RM is distributed to final users together with a certificate declaring its characteristics (including limitations such as cross-reactivity or inapplicability for certain methods) when it becomes a certified RM (CRM). Documents published by the International Organization for Standardization (ISO) outline theory and guidelines for the application of RMs. One of them is ISO Standard 17,511:2003: Guidelines for calibrator traceability.

International agencies of high reputation or an interconnected network of laboratories decide which methods are suitable to become reference ones. Each method is characterized by analytical parameters: accuracy, sensitivity, precision, repeatability, reproducibility, measurement interval, analytical interference, possible cross-reactivity with homologous analytes present in the sample.

Analytical parameters can be defined and/or explained as follows:

• The measured value of an analyte should be the closest possible to the accepted reference value (accuracy).
• The method should measure as low quantities of an analyte as possible (sensitivity), in order to achieve early detection of changes, when intervention measures have a greater chance of success.
• A method should be precise and the obtained values for multiple probes scattered as little as possible around the target value. It is worth noting that measuring precisely is not necessarily measuring accurately (true value).
• The analyte in a single sample, assayed in several tests over a period of time (several days, weeks or months if stored appropriately), should be measured with the smallest possible variation in the result (repeatability).
• The smallest possible variation in the results should be obtained by measuring a single sample, by the same method, with identical assay components in different laboratories, on different equipment, by different operators (reproducibility).
• The measurement interval should cover the entire range of normal values plus values below and above, up to several times the reference limit. Each method, however, has its limitations. Above (and sometimes below) a certain concentration or activity, measured values deviate from the initial relation between the variable and the response. Thus, for each method, a range of application must be declared.
• Analytical interference is the effect caused by the influence of a sample component which does not itself produce a response in the measuring reaction, but causes an increase or decrease of the actual result (e.g. absorbs in the same range of the spectrum as the reaction product).
• As protein families exhibit some degree of structural homology, it is important to know the cross-reactivity of the reagents with similar analytes, especially if they cannot be removed before the analysis. Thus, the reference method should be defined in terms of cross-reactivity and recognition of homologous species.

Although not a parameter of the analytical method, but still very important for analytical performance of a test, is the matrix effect. It is an intrinsic property of a sample, which does not augment or depress the measuring signal, but influences the measuring process and the final value (e.g. sample viscosity) systematically and more in a technical manner.

## Quantification: why is it hard?

Methods for quantification of simple and small analytes are relatively easily and reliably standardized, such as those for determination of glucose, cholesterol, ions. It is a much harder task to standardize methods for quantification of complex and large analytes, such as proteins, in physiological fluids or cell/tissue samples [37]. There are many obstacles to the standardization and quality control pathways and some specific ones for PTMs are discussed here.

If the analytical aim is to measure the specific protein in its native form, the choice of methods is very limited. Applicability of the method in every-day routine measurement imposes a further limitation. So-called “high-throughput” techniques that can handle many samples in a relatively short period of time are demanded in clinical practice. Finally, the concept of “cost-effectiveness” also has to be taken into account. Therefore, considering all analytical, clinical and practical aspects of PTM quantification, the demands can be summarized as follows:

• Reliable identification of nePTM which are directly correlated to the specific modifying reaction or reactive species which causes it.
• High specificity of intra-molecular nePTM recognition (as little as possible cross-recognition with similar nePTM within the same protein).
• High specificity of inter-molecular nePTM recognition (as little as possible cross-recognition of the same nePTM in different proteins).
• High sensitivity of nePTM recognition (detection of minor modification, just a single one if possible).
• “Acceptable justification” concerning determination of specific nePTM in relation to the specific (patho)physiological disturbance.
• “High-throughput” method and the equipment to support it.

In the following paragraphs, problems in satisfying these demands are discussed.

## Immunochemical assays for quantification of nePTM proteins

Specific proteins are most often measured by immunochemical methods, as immunoassays have been shown to possess the most optimal combination of the requested characteristics listed above. In contrast to methods for determination of simple analytes, which are based on simple stoichiometric relation between reactants, immunochemical methods are based on conformational recognition and affinity-binding [38].

Protein standards and physiological samples do not necessarily contain the same molecules. Standards represent only “the most frequent” combination. Many proteins exist in several isoforms, which may be present in diverse ratios, contributing to both inter-individual and intra-individual differences [39]. Follow-up studies on healthy population have shown that intra-individual variations among proteins, both in terms of quantity and isoform frequency, may be as much as 30% in a relatively short period of time (several months and even several weeks) [40]. The situation becomes more complicated due to natural ageing or in the case of disease, introducing new molecular forms.

Numerous modified derivatives of proteins are produced by the activity of many agents. Several modifications may occur within a single protein, some of which affect only one site and others multiple sites, forming an array of products. Many proteins give rise to an enormous number of variants. Two major problems in the determination of proteins are (i) how to recognize reliably the specific molecular protein form without interference by other, similar forms and (ii) how to recognize and measure a specific modification (or product) of a single protein without detecting the same modification present on another protein. Up to now, results have been only partially satisfactory.

The nePTM products are often unstable and degrade or change further immediately after sampling [41]. In order to be measured, these derivatives need to be relatively stable. When we consider relative stability, this means that specific protein forms are stable for at least a few hours, which is the minimum time needed for sample preparation and assay performance.

Confidence concerning standardization is not equal for all methods and may depend on technique. Even assay buffer content has been demonstrated to influence the measurement [42]. The presence of detergents may be necessary for complete solubilization of the standard or RM, but it may interfere with the determination in some methods.

Sensitivities of different methods vary several-fold. Physiological samples may be assayed directly in some methods but in others they must be diluted before being used (very often). Any manipulation with a sample can cause change in the nePTM, giving an inaccurate result. A standard or RM should preferably have a concentration/activity in the middle of the detection range for a specific method. As analytical ranges of different methods also vary several-fold, an alternative solution for standardization and quality control is production of a set of standards or RMs, with different concentrations or activities.

Assay response should be directly proportional to the amount of nePTM, but that is the case only when all modifications are available (i.e. exposed at the protein surface). If they are not, their “visibility” by the reagent depends on the conformational availability. In the previous chapter on folding and binding properties of proteins, we described variables that influence the conformation of a protein. Exposure of the specific PTM to be detected and measured depends on many factors, environmental ones being among the most important.

In the field of nePTM proteins there are no (C)RMs except for glycated hemoglobin (HbA1c), and there are no internationally accepted reference method(s). The absence of international standardization has initiated national standardization organizations to establish national reference methods. A step further in standardization of HbA1c quantification was made by the International Federation of Clinical Chemistry and Laboratory Medicine (IFCC) Work Group which announced that reference measurement procedures for HbA1c are HPLC–MS or HPLC–CE [43].

The general lack of RMs and/or standards has resulted in research based on nePTM preparations produced mostly “in house”, together with a range of experimental data for the same subject, some of which is in disagreement. An additional consequence of non-existent RMs is variation in analytical characteristics and performance of commercial assays for quantification of PTMs, as manufacturers of in vitro diagnostic tests (IVD tests) apply their own standardization procedures using their own standards.

## Spectrometric methods for quantification of nePTM proteins

Besides immunoassays, other methods are used for determination of nePTM, but not many are suitable for nePTM quantification. Spectrophotometric methods are employed for the measurement of one particular nePTM on all proteins in a sample. Other spectrometric methods, such as Raman and nuclear magnetic resonance (NMR) spectroscopy can identify specific nePTM and their positions within the molecule, but they need an isolated protein. Mass spectrometry (MS) is assumed to be the most precise method for detection and location of a specific modification. Since oxidative protein modifications are the most common nePTM related to ageing and disease, standardization of their quantification by MS will be discussed in more detail.

MS is an important tool in the discovery and characterization of nePTM proteins. It allows for accurate mapping of modification sites in the primary structure of a protein. It can also be used for quantifying the abundance of the peptide carrying the modified residue and to estimate either its modification level in relation to the unmodified peptides or between two or more different biological conditions. Thus, MS appears to be an ultimate tool that should be used for characterizing protein modification standards.

The unique feature of MS is that it can be used to analyze any protein modification without a priori assumptions about the type of modification. Based on the mass difference between the genome deduced protein sequence and peptide masses observed experimentally it is possible to identify any protein modification (reviewed in [44]). For example, oxidation of a Met residue (131 Da) increases its mass to 147 Da by the addition of single oxygen atom (16 Da). Through observation of the discrete mass difference of the intact protein or peptide it is possible to assign the respective modification. Additionally, mass spectrometry based sequencing allows for site-specific assignment of modifications at the resolution of individual amino-acids [45], [46], [47].

Although modern mass spectrometry instruments have exceptional sensitivity and can detect peptides at sub-femtomole levels [48], investigation of protein nePTM is still an analytical challenge (for a review, see [49]). This is mainly because modified proteins occur at very low levels in cells. Therefore, modification of a given site is often present in only a small fraction of the protein molecules of a given type. This phenomenon is also true for oxidized proteins.

Mass spectrometry relies on ionization of the peptides that are measured. In positive-ion operating mode, ionization of peptides strongly depends on the presence of basic sites like N-terminal amine and the side groups of Lys, Arg and His residues. Generation of some modifications, e.g. carbonyl products of Arg and Lys (to give glutamic and 2-amino-adipic semialdehydes) is associated with loss of guanidine and ammonia groups, typically carrying a positive charge. This leads to a decreased ionization efficiency and reduces the chance of detecting those peptides using positive-ion mode MS.

What makes analysis of oxidized proteins exceptionally challenging, compared to analysis of phosphorylated or acetylated proteins, for example, is the existence of many types of oxidative modifications of proteins. One of the most diverse oxidative modifications is carbonylation. Carbonylated residues can occur on amino-acid side chains, or can reside on the N-terminus of a cleaved protein; they can also be introduced into proteins by conjugation with oxidized lipids and carbohydrates. A comprehensive list of carbonylated residues can be found in [50]. Apart from carbonylation many other oxidative modifications can be introduced into different amino-acids. These may co-exist in oxidized proteins together with carbonylated residues [50].

The technical challenges related to the identification of protein nePTM have led scientists to develop specific strategies that allow the analysis to be focused on a particular group or type of protein modification. This is typically achieved via specific enrichment and/or chemical derivatization methods that target a certain class of modifications. This creates a “handle” by which modified peptides/proteins can be pooled out from the bulk of non-modified ones. Examples of analytical strategies aimed at nePMT enrichment are use of TiO_2_ for phosphorylated proteins [49], [51], use of biotin hydrazide for carbonylated proteins [50] or use of iodo-TMT to label and enrich proteins carrying oxidized Cys residues [52].

NePTM can also be analyzed by MS in “bottom up” experiments that rely on sample proteolysis prior to mass spectrometric detection or in “top-down” experiments that analyze intact proteins. The top down experiments provide more information about individual proteins, including full characterization of each protein form present and its modifications [53]. However, top-down proteomics is a relatively immature field compared to bottom-up proteomics, and still suffers from serious limitations [54].

Comprehensive interpretation of MS data obtained from nePTM focused experiments is an additional analytical challenge. The complexity of the data and limitations of the existing tools for bioinformatics very often lead to misinterpretation of the results. For example, modification of Lys and Arg residues frequently makes them inaccessible for trypsin proteolysis leading to a large number of missed cleavages. The resulting peptides require large numbers of missed cleavages to be included in data analysis algorithms increasing the chance of false positive identifications. Another difficulty comes from the many different modifications that need to be included. Searching algorithms (e.g. Mascot) often cannot search with an unlimited number of modifications, necessitating several searches of the same data. This increases data processing time and the risk of false positive identifications. New types of modifications can only be found if manual data analysis is carried out, but this approach is labor intensive, time consuming and cannot be used effectively with complex protein mixtures. Identification of oxidized peptides with a search algorithm poses several other technical problems. For detailed discussion of bioinformatics challenges in identification of carbonylated residues please see the review [50].

In spite of the difficulties in identifying carbonylation sites, the combination of labeling and enrichment methods described above has led to the identification of several hundred carbonylation sites in specific proteins [50].

Modern protein mass spectrometry techniques are used both to detect nePTM and to quantify their amount. This can be done either as relative measurement defining the difference between two conditions, e.g. diseased versus control sample or as an absolute measurement. Combined measurements of modified and unmodified forms of a particular peptide allow the percentage site occupancy to be defined, i.e. the number of modified molecules in relation to the number of unmodified molecules [52].

The most commonly used quantitative strategy in proteomics is relative quantitation. This can be achieved using several different techniques, for example the stable isotope labeling by amino acids in cell culture (SILAC) approach [55], [56], various chemical labeling procedures, e.g. trypsin-catalyzed ^18^O labeling [57] or dimethyl labeling [58], [59] or by isobaric labeling, i.e. Isobaric tag for relative and absolute quantitation (iTRAQ) and Tandem mass tag (TMT). Absolute quantitation of peptides and proteins is used to determine exact amounts of the analyzed samples. This is often achieved by spiking known amounts of heavy-labeled standards into the sample prior to LC–MSMS analysis and subsequently comparing the intensities of such standards and the analyte. Examples of this approach are AQUA [60] and QconCAT [61], which utilize isotope labeled peptides.

Multiple reactions monitoring (MRM) or single or selected reaction monitoring (SRM) is a targeted MS based quantitative approach. This technique allows measurement of the quantity of specific peptides of interest, selected according to their m/z and fragment ions generated upon MS–MS fragmentation. The resulting fragment ions confirm the identity of the precursor and their intensity is proportional to its abundance. This technique is often described as “large scale Western blotting” because it allows for simultaneous quantification of up to 100 proteins in one LC MS-SRM experiment [62]. Use of synthetic isotope labeled peptide equivalents as internal standards enables relative or absolute quantitation in SRM experiments. Unfortunately, most oxidative modifications are not available through commercial sources of synthetic peptides. Nevertheless, SRM has the potential to be a powerful technique for monitoring oxidative modifications [63]. Comprehensive reviews of different MS based quantification methods can be seen in [64], [65], [66].

It is very difficult to obtain inter-laboratory standardization in the analysis of modified proteins. This is due to the existence of a wide variety of instrumental and experimental setups. Over recent years scientists have become aware of this drawback and now acknowledge the necessity of standardization and unification. International initiatives have been undertaken during the last few years. The most prominent is The Human Proteome Organization (HUPO) Proteomics Standard Initiative (PSI). Its major focus is standardization of large scale proteomics experiments to facilitate data validation, accessibility and experimental transparency [67]. As an example of such work it has become a quality standard to use phosphoRS or similar algorithms to evaluate the probabilities associated with the precise localization of phosphorylation sites in peptide sequences [68].

In summary, the range of mass spectrometry based tools for characterization of protein nePTM is broad. All of these tools have strong points and limitations (reviewed in [65], [69], [70], [71]). To take full advantage of MS technology the experimental aims and the instrumentation at hand should be carefully considered.

## Commercial and “in house” made non-enzymatically modified proteins

Nowadays it is possible to synthesize peptides with specific PTM commercially. This approach is widely used in the field of phosphoproteomics, but other modifications can also be synthesized. Unfortunately, this does not work so well with protein oxidation for many reasons, e.g. stability and cross reactivity of the products. In the case of carbonyls some pseudo-aldehydes can be used [72].

For the purpose of this article, commercially available modified proteins were sought. Several producers of PTM proteins and also a number of products were found. We use the terms “product” or “preparation” instead of “standard”, as these items (except HbA1c from one producer) have not been officially recognized as standards or RMs. Some commercially available modified proteins are listed in Table 1. As can be seen, bovine serum albumin (BSA) is the major substrate used to produce PTM protein. Also, four different PTM albumin products can be found on the market: nitrated, glycated, carboxylmethyl-lysine and carbonylated. Thus, according to the manufacturers’ catalogs, the available PTM proteins are produced most likely under a standardized regime and defined as stable preparations with declared characteristics.

**Table 1:** Commercially available modified proteins.

**Modified protein**	**Producers**
Glycated human serum albumin	Sigma Aldrich, Exocell Inc.
Glycated human hemoglobin	Sigma Aldrich (Fluka), Exocell Inc.
Nitrated bovine serum albumin	Sigma Aldrich, Cell Biolabs Inc., Gentaur Molecular Products, Academy Bio-medical Co.
Glycated bovine serum albumin	Sigma Aldrich, BioVision Inc.
Carboxylmethyl-lysine bovine serum albumin	Cell Biolabs Inc., Academy Bio-medical Co.
Carbonylated bovine serum albumin	Cell Biolabs Inc.
Nitrated ovalbumin	Gentaur Molecular Products

Again, for the purpose of this article, we screened the scientific literature (approximately 150 papers published between 2000 and 2014), looking for data on the nePTM proteins employed as standards and discovered that most researchers do not use commercial modified proteins, but “in house” made preparations. Commercial nePTM proteins were quoted in less than 5% of the reports. Analysis of the frequency of appearance of “in house” made preparations revealed that most researchers modify albumin. Modifications of ovalbumin, apolipoproteins, collagen, immunoglobulins and β-amyloid were reported in several papers each, while there were sporadic cases when other proteins were modified to serve as standards. A list of “in house” prepared nePTM proteins to be used as standards is given in Table 2.

**Table 2:** “In house” made nePTM proteins.

**Modified protein**	**Frequency of application (%)**
Serum albumin (BSA+HSA+rodent SA)	50+16+7=73
Ovalbumin	3
Apolipoproteins	4
Collagen	5
Immunoglobulins	3
Beta-amyloid	5
Others (insulin, papain, myoglobin, RNAse, lactoglobulin, hemoglobin, crystalline, various enzymes, membrane proteins)	7

A further literature search led us to conclude that the majority of researchers investigate protein (glyco, lipo)oxidation. Therefore, we turned back to the datasheets which accompany commercial oxidized proteins aiming to investigate the characteristics and application opportunities of each preparation. The initial idea was to compare them. Unfortunately, that could not be done, as the available data accompanying the products differed. Here is a collection of information announced by different producers: (i) number of modified residues per molecule of protein, (ii) identity of the reactive species used for modification, (iii) the change in fluorescence of the modified molecule compared to the native one, (iv) the method in which the product can be used. Introduction of the standardization concept in “standard” production and characterization will certainly increase the applicative potential of commercial nePTM proteins.

Obviously, there is a need for reliable standards, produced and tested under strict conditions and precisely characterized in respect to the type of PTM, the quantity and, if possible, the position within the relevant protein molecule. As previously mentioned, there is only one CRM in the field of nePTM proteins and that is glycated human hemoglobin IRMM IFCC466. In their product datasheets manufacturers of IVD tests for quantification of HbA1c declare that they use this CRM to assign values for their calibrators which are prepared from pooled human erythrocytes. Manufacturers of immunoassays for quantification of HbA1c have even adopted identical test procedures in respect to concentrations and volumes of the reactants, times of incubation and wavelength to record the absorbance. A laboratory investigation (in INEP) on HbA1c immunoassay kits sold by three producers (Human, Germany; Elitech, France and Point Scientific, USA) demonstrated complete cross-reactivity of the calibrators in three assays and overlapping of standard curves (data not shown). In other words, the calibrators from one assay kit can be used for calibration of the assay produced by another manufacturer.

## Strategy to implement standardization and quality control in quantification of protein modifications

Standardization and quality control are necessary elements in quantification of PTM proteins. Since there are two aspects of PTM protein quantification, each standardization needs to be handled separately. If the idea is to quantify certain PTM which may be present on many proteins, a common standard possessing the particular PTM may be used. If the idea is to quantify a certain PTM on a specific protein, the standard can be that same protein modified to express that particular PTM. Alternatively, for the second purpose, the specific protein may be isolated and then measured against a common protein standard which has the appropriate PTM. In either case, the first step is to define suitable standard(s).

It is reasonable to choose a standard among already existing commercial products instead of creating new one(s), as manufacturers have probably already solved practical problems concerning continuous production of PTM proteins, their stabilization and storage. The next step is to network research laboratories and IVD test manufacturers to participate in inter-laboratory testing in order to determine the suitability of standards for different methods, sample types and purposes. Inter-laboratory (ring) testing will display the strengths and weaknesses of each method.

The most difficult analytical task dependent on the quality of standardization is detection and accurate quantification of minor proteins in complex (physiological) specimens. Since one specific nePTM protein is most often a minor fraction in the entire population of that protein, the detection range of the nePTM molecule is several fold lower than for the entire protein. As no official standardization in the field of PTM proteins exists except for HbA1c, it is reasonable to start with standardization and quality control in quantification of specific nePTM present on all (or many) proteins in a chosen sample, as an easier task.

Once the first inter-laboratory testing is completed and data analyzed, the final result will most probably point to significant differences between methods and laboratories. According to the experience from some ring trials, quite often no firm conclusions could be drawn regarding the optimal method, as participants using same method did not obtain comparable results. The most experienced laboratories submitted the most accurate results. Thus, successful protein quantification requires a combination of method, equipment and experienced operators. Further adjustments in assay procedures and subsequent inter-laboratory trials will lead to more encouraging results and, hopefully, to decisions on potential standard(s), RM(s) and reference method(s).

A multi-center study was conducted in six laboratories across Europe to validate the measurement of protein carbonyls (PCO) [73]. ELISA and Western immunoblotting methods were employed to detect differences in the amount of PCO in protein samples exposed to UV irradiation for different time intervals. The results demonstrated that all laboratories measured increased PCO content in the sample after 5 min of UV irradiation compared to the native sample. However, half of the laboratories detected less PCO after protein irradiation for 15 min than for 5 min. It was suggested that the reason for such diversity may be found in errors in calculating the amount of PCO, due to differences in method standardization in each laboratory and, to some extent, in procedural differences (i.e. proteolytic cleavage of oxidized proteins). The authors concluded that highly oxidized proteins were not effectively measured by the techniques tested in the study.

Since no method is perfect and no standard will equally suit all methods, some consensus decisions have to be made. The most important concern the assessment of measurement inaccuracy and uncertainty. If the test is standardized to have very high sensitivity, it may record false negative results. If the test is standardized to have relatively low specificity, it may give false positive results.

Measurement uncertainty is a variable characteristic for a laboratory performing tests. It provides quantitative estimation of the level of confidence that the particular laboratory is measuring precisely. Measurement uncertainty (MU) is not a component of assay standardization, but it is an integral part of quality control. The implementation of MU is described in ISO Standard 15,189:1995: Guide to the expression of uncertainty in measurement. This was written primarily for laboratories performing classical physico-chemical measurements. Approaches to implement MU standards in the measurement of large physiological molecules, such as proteins, are still being developed.

Following consensus decisions and official recognition of standards and/or RMs, together with acceptable and/or reference methods, research and clinical laboratories, as well as IVD manufacturers, are obliged to apply official guidelines in standardization and quality control in nePTM protein quantification. Additionally, implementation of standardization and quality control in test manufacture will lead to unification in analytical characteristics and performance of commercial IVD assays.

Standardization and quality control are instruments to achieve comparability of nePTM protein quantification results over time and space, enabling definition of decision-making criteria that can be used in health assessment in relation to ageing and diseases.

To conclude, in order to standardize the quantification of certain nePTM proteins, we propose the following:

• Creation of a formal group responsible for standardization (suggestion: from appropriate scientific societies, IVD producers, possibly from stakeholders responsible for health).
• The choice of one commercial nePTM protein to be treated as the standard (suggestion: the nePTM most often studied, i.e. carbonylation; the protein most often determined, i.e. albumin; from a manufacturer with stable production of the specific material over several years).
• Networking as many laboratories as possible to participate in a standardization trial (suggestion: open call via scientific societies; including producers of commercial IVD assays; optional, to see whether the producer of the chosen nePTM protein is interested in participation, which may lower the cost of the trial).
• Distribution of the material together with instructions and form (suggestion: the form should contain questions both on technical aspects of method/instrument and the experience of the laboratory; perhaps “on-line” form).
• Data collection and analysis.
• Decision on outcomes of standardization: applicability of the chosen nePTM protein as the standard for the methods used, intra- and inter-assay variations, determination of standard stability, selection of most reliable laboratory(s).
• Decision on participants for quality control trial (suggestion: definition of inclusion criteria based on the results of the standardization trial)
• Distribution of the quality control sample(s) with instructions and form (suggestion: 1–3 samples, could be either commercial or made in a reliable laboratory and tested for stability).
• Data collection and analysis.
• Decision on outcomes of the quality control testing: applicability of the quality control samples for the methods used, confirmation of the most reliable laboratories.
• Final decisions: (i) is the commercial nePTM protein used suitable to be termed “standard” and (ii) are the quality control samples used suitable to be officially termed “quality control (or proficiency testing) samples”?
• Publication of results, dissemination of conclusions, organization of proficiency testing scheme, implementation of standardization in production of commercial IVD assays.

## Conflicts of interest

The authors declare that there are no known conflicts of interest associated with this publication.

## References

[1] Kumar S., Ma B., Tsai C.-J., Sinha N., Nussinov R. (2000). Folding and binding cascades: dynamic landscapes and population shifts. Protein Science.

[2] Ma B., Shatsky M., Wolfson H.J., Nussinov R. (2002). Multiple diverse ligands binding at a single protein site: a matter of pre-existing populations. Protein Science.

[3] Uversky V.N. (2013). Under-folded proteins: Conformational ensembles and their roles in protein folding, function, and pathogenesis. Biopolymers.

[4] Vandermarliere E., Mueller M., Martens L. (2013). Getting intimate with trypsin, the leading protease in proteomics. Mass Spectrometry Reviews.

[5] Chuenchor W., Jin T., Ravilious G., Xiao T.S. (2014). Structures of pattern recognition receptors reveal molecular mechanisms of autoinhibition, ligand recognition and oligomerization. Current Opinion in Immunology.

[6] Schreiber G., Keating A.E. (2011). Protein binding specificity versus promiscuity. Current Opinion in Structural Biology.

[7] Bhardwaj N., Abyzov A., Clarke D., Shou C., Gerstein M.B. (2011). Integration of protein motions with molecular networks reveals different mechanisms for permanent and transient interactions. Protein Science.

[8] Nakamura A., Kawakami K., Kametani F., Nakamoto H., Goto S. (2010). Biological significance of protein modifications in aging and calorie restriction. Annals of the New York Academy of Science.

[9] Šoškić V., Groebe K., Schrattenholz A. (2008). Nonenzymatic posttranslational protein modifications in ageing. Experimental Gerontology.

[10] Höhn A., König J., Grune T. (2013). Protein oxidation in aging and the removal of oxidized proteins. Journal of Proteomics.

[11] Han S.-H., Kim Y.H., Mook-Jung I. (2011). RAGE: the beneficial and deleterious effects by diverse mechanisms of actions. Molecular Cell.

[12] Stevens R.C., Sancho J., Martinez A. (2010). Rescue of misfolded proteins and stabilization by small molecules. Methods in Molecular Biology.

[13] Lotz G.P., Legleiter J. (2013). The role of amyloidogenic protein oligomerization in neurodegenerative disease. Journal of Molecular Medicine.

[14] Shacter E., Williams J.A., Lim M., Levine R.L. (1994). Differential susceptibility of plasma proteins to oxidative modification: examination by western blot immunoassay. Free Radical Biology and Medicine.

[15] Lionaki E., Tavernarakis N. (2013). Oxidative stress and mitochondrial protein quality control in aging. Journal of Proteomics.

[16] Sawabe M. (2010). Vascular aging: from molecular mechanism to clinical significance. Geriatrics and Gerontolology International.

[17] Hipkiss A.R. (2006). Accumulation of altered proteins and ageing: causes and effects. Experimental Gerontology.

[18] Rattan S.I. (2008). Increased molecular damage and heterogeneity as the basis of aging. Biological Chemistry.

[19] Levine R.L. (2002). Carbonyl modified proteins in cellular regulation, aging, and disease. Free Radical Biology and Medicine.

[20] Cloos P.A., Christgau S. (2004). Post-translational modifications of proteins: implications for aging, antigen recognition, and autoimmunity. Biogerontology.

[21] Rattan S.I. (2010). Synthesis, modification and turnover of proteins during aging. Advances in Experimental Medicine and Biology.

[22] Ahmed E.K., Rogowska-Wrzesinska A., Roepstorff P., Bulteau A.L., Friguet B. (2010). Protein modification and replicative senescence of WI-38 human embryonic fibroblasts. Aging Cell.

[23] Carney J.M., Starke-Reed P.E., Oliver C.N., Landum R.W., Cheng M.S., Wu J.F., Floyd R.A. (1991). Reversal of age-related increase in brain protein oxidation, decrease in enzyme activity, and loss in temporal and spatial memory by chronic administration of the spin-trapping compound N-tert-butyl-alpha-phenylnitrone. Proceedings of the National Academy Sciences of the United States of America.

[24] Mary J., Vougier S., Picot C.R., Perichon M., Petropoulos I., Friguet B. (2004). Enzymatic reactions involved in the repair of oxidized proteins. Experimental Gerontology.

[25] Crabb J.W., Miyagi M., Gu X., Shadrach K., West K.A., Sakaguchi H., Kamei M., Hasan A., Yan L., Rayborn M.E., Salomon R.G., Hollyfield J.G. (2002). Drusen proteome analysis: an approach to the etiology of age-related macular degeneration. Proceedings of the National Academy Sciences of the United States of America.

[26] Nowotny K., Jung T., Grune T., Höhn A. (2014). Accumulation of modified proteins and aggregate formation in aging. Experimental Gerontology.

[27] Halliwell B. (2009). The wanderings of a free radical. Free Radical Biology and Medicine.

[28] Nedić O., Rattan S.I.S., Grune T., Trougakos I.P. (2003). Molecular effects of advanced glycation end products on cell signalling pathways, ageing and pathophysiology. Free Radical Biology and Medicine.

[29] Kueper T., Grune T., Prahl S., Lenz H., Welge V., Biernoth T., Vogt Y., Muhr G.-M., Gaemlich A., Jung T., Boemke G., Elsässer H.-P., Wittern K.-P., Wenck H., Stäb F., Blatt T. (2007). Vimentin is the specific target in skin glycation. Structural prerequisites, functional consequences, and role in skin aging. Journal of Biological Chemistry.

[30] Meli M., Frey J., Perier C. (2003). Native protein glycoxidation and aging. Journal of Nutrition Health and Aging.

[31] Makita Z., Vlassara H., Rayfield E., Cartwright K., Friedman E., Rodby R., Cerami A., Bucala R. (1992). Hemoglobin-age: a circulating marker of advanced glycosylation. Science.

[32] Atli T., Keven K., Avci A., Kutlay S., Turkcapar N., Varli M., Aras S., Ertug E., Canbolat O. (2004). Oxidative stress and antioxidant status in elderly diabetes mellitus and glucose intolerance patients. Archives of Gerontology and Geriatrics.

[33] Yan L.-J., Levine R.L., Sohal R.S. (1997). Oxidative damage during aging targets mitochondrial aconitase. Proceedings of the National Academy Sciences of the United States of America.

[34] Panteghini M. (2009). Traceability as a unique tool to improve standardization in laboratory medicine. Clinical Biochemistry.

[35] Armbruster D., Miller R.R. (2007). The Joint Committee for Traceability in Laboratory Medicine (JCTLM): a global approach to promote the standardisation of clinical laboratory test results. Clinical Biochemistry Reviews.

[36] Bock J.L., Eckfeldt J.H. (2011). Advances in standardization of laboratory measurement procedures: implications for measuring biomarkers of folate and vitamin B-12 status in NHANES. American Journal of Clinical Nutrition.

[37] Schimmel H., Zegers I., Emons H. (2010). Standardization of protein biomarker measurements: Is it feasible?. Scandinavian Journal of Clinical and Laboratory Investigation.

[38] Masseyeff R.F. (1989). Factors limiting the comparability of the assay of antigens and antibodies. Scandinavian Journal of Clinical and Laboratory Investigation.

[39] Ellis J.M., Frahm J.L., Li L.O., Coleman R.A. (2010). Acyl-coenzyme A synthetases in metabolic control. Current Opinion in Lipidology.

[40] Erden G., Barazi A.O., Tezcan G., Yildirimkaya M.M. (2008). Biological variation and reference change values of CA 19-9, CEA, AFP in serum of healthy individuals. Scandinavian Journal of Clinical and Laboratory Investigation.

[41] Kubota K., Nakayama A., Takehana K., Kawakami A., Yamada N., Suzuki E. (2009). A simple stabilization method of reduced albumin in blood and plasma for the reduced/oxidized albumin ratio measurement. International Journal of Biomedical Science.

[42] Uludagˇ Y., Tothill I.E. (2010). Development of a sensitive detection method of cancer biomarkers in human serum (75%) using a quartz crystal microbalance sensor and nanoparticles amplification system. Talanta.

[43] John G., English E. (2012). IFCC standardised HbA(1c): should the world be as one?. Clinical Chemistry and Laboratory Medicine.

[44] Larsen M.R., Trelle M.B., Thingholm T.E., Jensen O.N. (2006). Analysis of posttranslational modifications of proteins by tandem mass spectrometry. BioTechniques.

[45] Mann M., Jensen O.N. (2003). Proteomic analysis of post-translational modifications. Nature Biotechnology.

[46] Jensen O.N. (2004). Modification-specific proteomics: characterization of post-translational modifications by mass spectrometry. Current Opinion in Chemical Biology.

[47] Jensen O.N. (2006). Interpreting the protein language using proteomics. Nature Reviews Molecular Cell Biology.

[48] Han X., Aslanian A., Yates J.R. (2008). Mass spectrometry for proteomics. Current Opinion in Chemical Biology.

[49] Zhao Y., Jensen O.N. (2009). Modification-specific proteomics: strategies for characterization of post-translational modifications using enrichment techniques. Proteomics.

[50] Møller I.M., Rogowska-Wrzesinska A., Rao R.S. (2011). Protein carbonylation and metal-catalyzed protein oxidation in a cellular perspective. J. Proteomics.

[51] Thingholm T.E., Jensen O.N., Larsen M.R. (2009). Analytical strategies for phosphoproteomics. Proteomics.

[52] Wojdyla K., Williamson J., Roepstorff P., Rogowska-Wrzesinska A. (2015). The SNO/SOH TMT strategy for combinatorial analysis of reversible cysteine oxidations. J. Proteomics.

[53] Siuti N., Kelleher N.L. (2007). Decoding protein modifications using top-down mass spectrometry. Nature Methods.

[54] Chait B.T. (2006). Chemistry. Mass spectrometry: bottom-up or top-down?. Science.

[55] Ong S.-E., Blagoev B., Kratchmarova I., Kristensen D.B., Steen H., Pandey A., Mann M. (2002). Stable isotope labeling by amino acids in cell culture, SILAC, as a simple and accurate approach to expression proteomics. Molecular and Cellular Proteomics.

[56] Mann M. (2006). Functional and quantitative proteomics using SILAC. Nature Reviews Molecular Cell Biology.

[57] Mirgorodskaya O.A., Kozmin Y.P., Titov M.I., Körner R., Sönksen C.P., Roepstorff P. (2000). Quantitation of peptides and proteins by matrix-assisted laser desorption/ionization mass spectrometry using (18)O-labeled internal standards. Rapid Communications in Mass Spectrometry.

[58] Hsu J.-L., Huang S.-Y., Chow N.-H., Chen S.-H. (2003). Stable-isotope dimethyl labeling for quantitative proteomics. Analytical Chemistry.

[59] Boersema P.J., Raijmakers R., Lemeer S., Mohammed S., Heck A.J. (2009). Multiplex peptide stable isotope dimethyl labeling for quantitative proteomics. Nature Protocol.

[60] Gerber S.A., Rush J., Stemman O., Kirschner M.W., Gygi S.P. (2003). Absolute quantification of proteins and phosphoproteins from cell lysates by tandem MS. Proceedings of the National Academy of Sciences of the United States of America.

[61] Beynon R.J., Doherty M.K., Pratt J.M., Gaskell S.J. (2005). Multiplexed absolute quantification in proteomics using artificial QCAT proteins of concatenated signature peptides. Nature Methods.

[62] Picotti P., Aebersold R. (2012). Selected reaction monitoring-based proteomics: workflows, potential, pitfalls and future directions. Nature Methods.

[63] Madian A.G., Myracle A.D., Diaz-Maldonado N., Rochelle N.S., Janle E.M., Regnier F.E. (2011). Differential carbonylation of proteins as a function of in vivo oxidative stress. Journal of Proteome Research.

[64] Bantscheff M., Schirle M., Sweetman G., Rick J., Kuster B. (2007). Quantitative mass spectrometry in proteomics: a critical review. Analytical and Bioanalytical Chemistry.

[65] Bantscheff M., Lemeer S., Savitski M.M., Kuster B. (2012). Quantitative mass spectrometry in proteomics: critical review update from 2007 to the present. Analytical and Bioanalytical Chemistry.

[66] Wasinger V.C., Zeng M., Yau Y. (2013). Current status and advances in quantitative proteomic mass spectrometry. International Journal of Proteomics.

[67] Hermjakob H. (2006). The HUPO proteomics standards initiative—overcoming the fragmentation of proteomics data. Proteomics.

[68] Taus T., Köcher T., Pichler P., Paschke C., Schmidt A., Henrich C., Mechtler K. (2011). Universal and confident phosphorylation site localization using phosphors. Journal of Proteome Research.

[69] Lau K.W., Jones A.R., Swainston N., Siepen J.A., Hubbard S.J. (2007). Capture and analysis of quantitative proteomic data. Proteomics.

[70] Miyagi M., Rao K.C. (2007). Proteolytic ^18^O-labeling strategies for quantitative proteomics. Mass Spectrometry Reviews.

[71] Treumann A., Thiede B. (2010). Isobaric protein and peptide quantification: perspectives and issues. Expert Review of Proteomics.

[72] Todorovski T., Fedorova M., Hennig L., Hoffmann R. (2011). Synthesis of peptides containing 5-hydroxytryptophan, oxindolylalanine, N-formylkynurenine and kynurenine. Journal of Peptide Science.

[73] Augustyniak E., Adam A., Wojdyla K., Rogowska-Wrzesinska A., Willetts R., Korkmaz A., Atalay M., Weber D., Grune T., Borsa C., Gradinaru D., Chand Bollineni R., Fedorova M., Griffiths H.R. (2015). Validation of protein carbonyl measurement: a multi-centre study. Redox Biology.

